# Microglia: roles and genetic risk in Parkinson’s disease

**DOI:** 10.3389/fnins.2024.1506358

**Published:** 2024-11-01

**Authors:** Alex R. Trainor, Debra S. MacDonald, Jay Penney

**Affiliations:** Department of Biomedical Sciences, AVC, University of Prince Edward Island, Charlottetown, PE, Canada

**Keywords:** Parkinson’s disease, microglia, genetic risk, neurodegeneration, neuroinflammation

## Abstract

The prevalence of neurodegenerative disorders such as Parkinson’s disease are increasing as world populations age. Despite this growing public health concern, the precise molecular and cellular mechanisms that culminate in neurodegeneration remain unclear. Effective treatment options for Parkinson’s disease and other neurodegenerative disorders remain very limited, due in part to this uncertain disease etiology. One commonality across neurodegenerative diseases is sustained neuroinflammation, mediated in large part by microglia, the innate immune cells of the brain. Initially thought to simply react to neuron-derived pathology, genetic and functional studies in recent years suggest that microglia play a more active role in the neurodegenerative process than previously appreciated. Here, we review evidence for the roles of microglia in Parkinson’s disease pathogenesis and progression, with a particular focus on microglial functions that are perturbed by disease associated genes and mutations.

## Introduction

Parkinson’s disease (PD) is the second most common neurodegenerative disease and the fastest growing neurological disorder (Bloem et al., 2021). While our understanding of the mechanisms that underly neuron dysfunction and death in PD have improved tremendously, this understanding remains far from complete (Müller-Nedebock et al., 2023). More troubling, there is a lack of effective treatment options for PD. While existing treatments can temporarily relieve symptoms, they are unable to halt neurodegeneration or alter the overall course of disease (Liu et al., 2022). There is, thus, an urgent need to more fully understand the mechanisms that contribute to neurodegeneration in PD and to facilitate the development of effective disease modifying therapeutics.

First described by James Parkinson more than 200 years ago, PD is a movement disorder characterized most prominently by four cardinal motor symptoms: slowness of movement (known as bradykinesia), postural instability, rigidity and tremor (Goetz, 2011). PD symptoms are caused by the loss of dopamine producing neurons from the substantia nigra pars compacta (SNpc) region of the midbrain (Przedborski, 2017). At the histopathological level most PD cases are characterized by the presence of Lewy bodies and Lewy neurites which are intraneuronal aggregates composed primarily of the protein α-Synuclein (Calabresi et al., 2023; Spillantini et al., 1997). In addition to facilitating motor control, SNpc dopaminergic (DA) neurons also regulate a number of other processes, thus motor symptoms in PD are frequently accompanied by cognitive, behavioral and autonomic disturbances (Bloem et al., 2021).

## Causes of Parkinson’s disease

There remains considerable debate about the most important causes of neurodegeneration in PD, as well as about the relative importance of genetic versus environmental factors (Müller-Nedebock et al., 2023; Lim and Klein, 2024; Dorsey and Bloem, 2024). While the majority of PD cases do not have an identified cause (i.e.,—are idiopathic), there is strong evidence that genetic and environmental factors are each capable of causing DA neuron degeneration in humans and model organisms (Ball et al., 2019; Vázquez-Vélez and Zoghbi, 2021). Importantly, those PD cases that do have a known cause provide an entry point for the identification of cellular and molecular mechanisms which can result in neurodegeneration, and which can be more broadly generalized to idiopathic PD.

It is widely thought that exposure to certain pesticides, industrial chemicals, air pollutants, heavy metals, and illicit drugs increase risk for PD, though establishing their causality unequivocally has been difficult (Dorsey and Bloem, 2024; Ball et al., 2019; Aravindan et al., 2024). Studies in model organisms have supported these epidemiological links, demonstrating that pesticides such as paraquat and rotenone, chemicals such as trichloroethylene, heavy metals such as iron and lead, and stimulants such as methamphetamine can lead to the degeneration of DA neurons in rodents (Ball et al., 2019; Graves et al., 2021; Ilieva et al., 2024). A key commonality among these environmental factors is their ability to generate reactive oxygen species (ROS) through various mechanisms (Ball et al., 2019; Graves et al., 2021; Ilieva et al., 2024). ROS aggressively target the electrons of numerous different cellular substrates, causing oxidative damage to proteins, lipids, and nucleic acids, resulting in oxidative stress and various forms of cellular dysfunction (Ni and Ernst, 2022). While the mechanisms are not fully understood, SNpc DA neurons appear to be unusually sensitive to the effects of ROS, in part accounting for their preferential loss in PD (Ni and Ernst, 2022). In the case of the pesticide rotenone, excessive ROS production is due to direct inhibition of complex I of the mitochondrial electron transport chain (Przedborski and Ischiropoulos, 2005). Mitochondrial complex I inhibition also underlies another intriguing and well documented cause of PD: the contamination of the synthetic opioid MPPP (1-methyl-4-phenyl-4-propionoxypiperidine) with MPTP (1-methyl-4-phenyl-1,2,3,6-tetrahydropyridine), which resulted in the development of PD in a subset of recreational MPPP users (Langston, 2017).

Genetic factors identified to impact PD span the range from highly penetrant mutations that cause familial forms of PD to low penetrance genetic risk factors that exert modest but statistically significant effects on the likelihood of developing disease (Lim and Klein, 2024; Vázquez-Vélez and Zoghbi, 2021). Roughly 15% of PD cases are familial, most being inherited due to high penetrance mutations in 7 different well-established PD genes: SNCA (encoding α-Synuclein), PRKN (encoding Parkin), PINK1 (encoding PTEN-induced putative kinase protein 1), PARK7 (encoding DJ-1), GBA1 (encoding Beta-glucosylceramidase 1), LRRK2 (encoding Leucine-rich repeat kinase 2), and VPS35 (encoding Vacuolar protein sorting-associated protein 35; Lim and Klein, 2024; Bandres-Ciga et al., 2020). High or moderate penetrance mutations in multiple other genes have also been identified as less frequent causes of PD or Parkinsonian phenotypes in the context of other syndromes (Bandres-Ciga et al., 2020; Day and Mullin, 2021). In more recent years, large-scale genome-wide association studies (GWAS) have allowed for the additional identification of close to 100 genetic risk loci where single nucleotide polymorphisms (SNPs) increase or decrease the likelihood of developing PD (Lim and Klein, 2024; Nalls et al., 2019). Altogether, it is estimated that polygenic risk for PD, which incorporates familial mutations together with genome-wide significant and sub-threshold risk loci, can account for almost 70% of the total risk for a person developing PD (Nalls et al., 2019).

Six of the seven high penetrance PD genes just listed have been known for 20+ years to be associated with disease (Bandres-Ciga et al., 2020). All have been extensively studied and together form much of the basis for our understanding of the cellular processes that can result in PD when perturbed (Vázquez-Vélez and Zoghbi, 2021; Bandres-Ciga et al., 2020). These processes include vesicular trafficking, lysosomal function, mitochondrial quality control, and immune functions, whose actions are often interrelated (Lim and Klein, 2024; Vázquez-Vélez and Zoghbi, 2021). Mutations in SNCA, identified in 1997, were the first identified genetic cause of PD, belying the central role that α-Synuclein plays in disease pathology (Polymeropoulos et al., 1997). Importantly, while α-synuclein aggregation and buildup are key pathological features shared across most familial and idiopathic cases of PD, α-Synuclein pathology can also impact each of the main cellular processes that have been implicated in PD, reinforcing the interrelated nature of these disease-associated mechanisms (Calabresi et al., 2023).

While more than 100 other genes and genetic loci have now been identified that can impact risk for PD, the large majority were identified in the last 5-10 years and have been far less studied than the high penetrance PD genes (Bandres-Ciga et al., 2020). It is not surprising, then, that the exact roles that most risk genes play in PD remains either uncertain or completely unknown. As such, elucidation of the perturbed cellular functions that link these PD risk genes to the development of disease is a major outstanding task for the field. The identification of such risk genes has, however, implicated additional pathways and processes in disease pathogenesis, and importantly, has also lent support to the idea that brain cell types beyond neurons are important in the development of PD (Vázquez-Vélez and Zoghbi, 2021; Kam et al., 2020). Indeed, across neurodegenerative diseases there is a growing appreciation that the functions of the different brain cell types are interconnected, with dysfunction of each cell type having the potential to promote neurodegeneration (Balusu et al., 2023). In particular, key roles for microglia have increasingly been established in the pathogenesis of multiple neurodegenerative diseases including PD (Gao et al., 2023).

## Microglia support neuronal health

**Figure 1:**
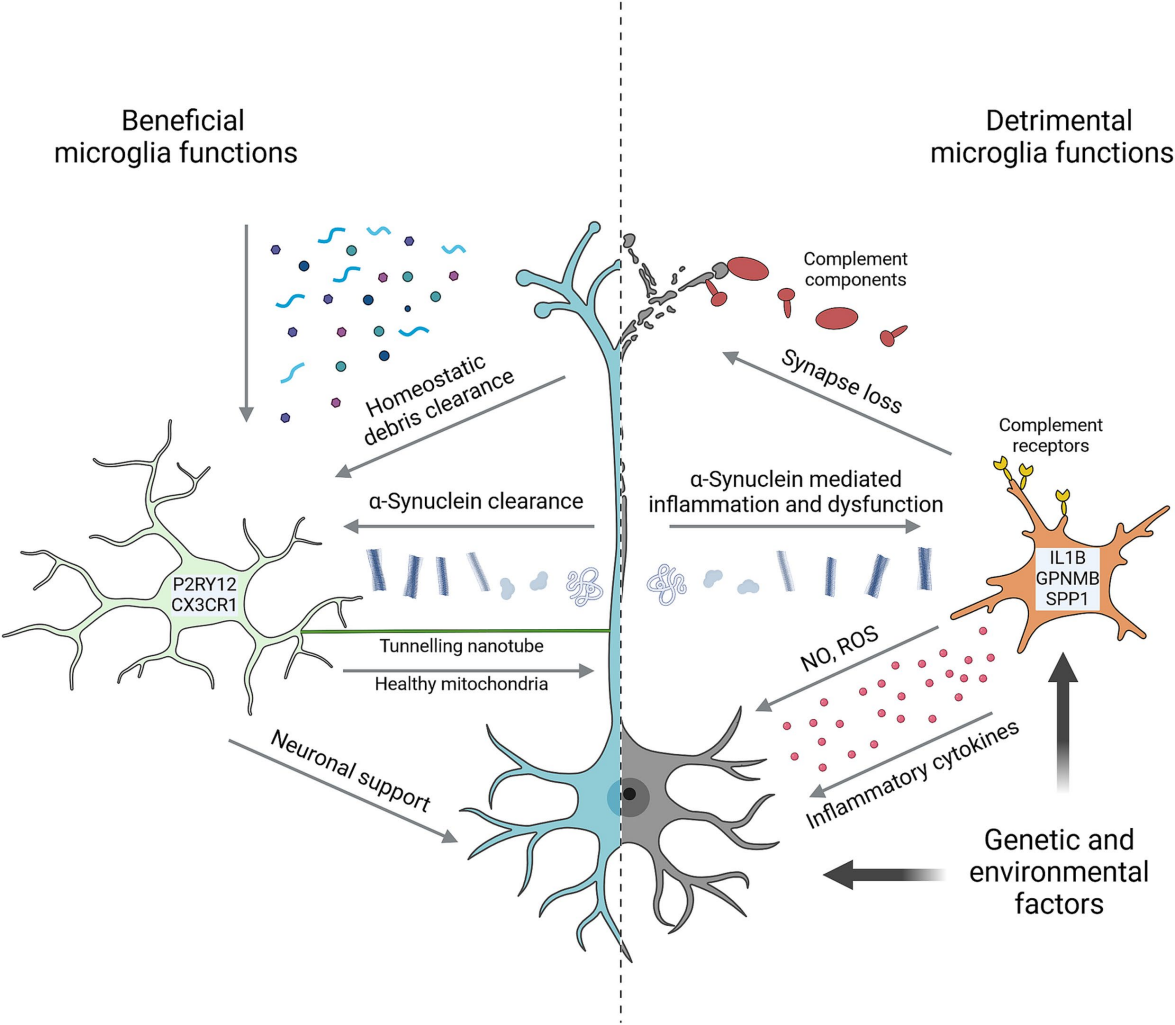
Key microglial functions in Parkinson’s disease. Microglia perform numerous homeostatic functions that support neuron health and counteract the development of disease. Characterized by high expression of markers such as P2RY12 and CX3CR1, they sense and remove a wide range of potentially detrimental substrates from the brain environment including various α-Synuclein species. Tunnelling nanotubes facilitate the direct intracellular transfer of α-Synuclein from neurons and healthy mitochondria to neurons. Trophic support and neuronal activity are other forms of support microglia provide to neurons. Microglia are also capable of harming neurons in multiple ways, including via neurotoxic cytokines, reactive oxygen species (ROS) and nitric oxide (NO), and by complement-mediated removal of synapses. In many cases, detrimental microglial effects are prompted by neuron-derived signals. α-Synuclein is prime among these, causing microglial inflammation and impairing homeostatic functions. Microglia in PD are often marked by high expression of genes such as IL1B, GPNMB and SPP1. Both genetic and environmental factors can impact neuron and/or microglial functions to promote neurodegeneration. Created with Biorender.com.

As the resident macrophages of the central nervous system, microglia perform numerous important functions in the developing, the adult, and the aging brain (Figure 1). Microglia actively survey their environment to sense and remove substrates including synaptic material, damaged myelin, protein aggregates, and components of dead cells to maintain ‘homeostasis’ of their brain environment (Gao et al., 2023; Li and Barres, 2018). Microglia can impact neurodevelopment and plasticity by providing trophic support, shaping neural circuits via synaptic pruning, as well as by sensing and regulating synaptic activity (Badimon et al., 2020; Salter and Stevens, 2017). Microglia are also the primary regulators of inflammation in the brain, mediating cytokine and chemokine release in response to a multitude of stimuli (Zhang et al., 2023).

These homeostatic roles continue to be important through adulthood and as we age, with microglia supporting the functions of neurons and other brain cells while also working to maintain the integrity of the brain environment (Li and Barres, 2018). More directly relevant to PD, microglia can internalize and degrade extracellular α-Synuclein, helping to counteract its harmful effects on brain cells and prevent pathological buildup (Gao et al., 2023). Microglia can also support neuron health via tunneling nanotubes (TNTs), which are thin, actin-rich, membrane-bound processes that can transport macromolecules, and even organelles, between cells (Rustom et al., 2004). TNTs between microglia and neurons have been shown to traffic both mitochondria and α-Synuclein aggregates, with microglia donating healthy mitochondria to neurons and accepting α-Synuclein for degradation (Scheiblich et al., 2024; Chakraborty et al., 2023).

## Microglia can damage neurons

Homeostatic microglial functions are critical for brain development and plasticity. However, persistent microglial activation results in chronic neuroinflammation that is a hallmark of neurodegenerative disease and can directly impact neuron health and survival (Figure 1; Gao et al., 2023; Zhang et al., 2023). In rodents, repeated systemic treatment with lipopolysaccharide (LPS), a pro-inflammatory bacterial cell wall component, can induce neuron degeneration that preferentially affects DA neurons (Neal et al., 2020). Like LPS, both monomeric and aggregated forms of α-Synuclein are strongly pro-inflammatory to microglia, and α-Synuclein injection or overexpression can similarly induce DA neurodegeneration (Gao et al., 2023; Thi Lai et al., 2024).

Consistent with the pro-inflammatory effects of α-Synuclein, brain imaging studies and postmortem gene expression profiling have frequently detected microglial activation and neuroinflammation in individuals with PD (Liu et al., 2022; Smajić et al., 2022; Wang Q. et al., 2024; Quan et al., 2024). Single cell and single nucleus sequencing studies have been central in identifying microglial sub-populations and gene expression changes that are associated with disease. Initial studies using mouse neurodegeneration models identified populations of ‘homeostatic’ and ‘disease associated’ microglia (DAM; Keren-Shaul et al., 2017; Krasemann et al., 2017). Homeostatic populations were marked by relatively high expression of genes such as Cx3cr1, P2ry12 and Tmem119, while prominent DAM population markers include Apoe, Gpnmb, Spp1 and Trem2 (Chen and Colonna, 2021). The mouse orthologs of a number of PD-associated/risk genes are also found among markers of both homeostatic (including DYRK1A, FAM49B and KPNA1) and DAM (including CTSB, GPNMB and VPS13C) populations (Nalls et al., 2019; Keren-Shaul et al., 2017). Single nucleus sequencing studies from PD patient postmortem samples show analogous microglial gene expression changes. These include reduced P2RY12 levels and increased GPNMB, IL1B and SPP1 expression, consistent with microglial activation and impaired homeostatic functions in the disease state (Smajić et al., 2022; Quan et al., 2024).

Microglial activation can damage DA neurons in multiple ways. Activated microglia can release an array of cytokines and chemokines, as well as producing ROS, nitric oxide and bioactive lipids (Zhang et al., 2023). These mediators can cause protein modifications, promote protein misfolding and potentially induce mitochondrial dysfunction in neighboring neurons (Zhang et al., 2023; Pirooznia et al., 2021). In addition, microglia and microglia-derived factors can promote the aggregation of α-Synuclein species (Pirooznia et al., 2021; Bartels et al., 2020). Chemokine and cytokine signaling from microglia can further promote blood brain barrier permeability and the recruitment of peripheral immune cells into the normally protected brain environment (Gao et al., 2023; Pirooznia et al., 2021). The inappropriate removal of synapses is another mechanism by which microglia can impact neuron health. Complement-mediated synaptic pruning by microglia is a key process involved in refinement of neural circuits during development. However, inappropriate re-activation of synaptic pruning has been observed in a number of neurodegenerative disease models and can contribute to neuron loss (Salter and Stevens, 2017; Penney et al., 2024; Lall et al., 2021; Wu et al., 2019).

Microglia-astrocyte crosstalk can also be important in neurodegeneration, whereby microglia-derived signals direct the conversion of astrocytes to a reactive phenotype that is toxic to neurons and oligodendrocytes (Liddelow et al., 2017; Lawrence et al., 2023). Both microglia-mediated synapse loss and astrocyte crosstalk have been implicated in the pathogenesis of PD models, with inhibition of reactive astrocyte conversion found to provide neuroprotection (Zhang et al., 2022; Yun et al., 2018). Importantly, depletion of microglia has also been shown to reduce the DA neurodegeneration induced by LPS and α-Synuclein treatments, indicating the key role of microglial inflammation in the neurodegenerative processes (Neal et al., 2020; Thi Lai et al., 2024).

## Parkinson’s disease pathomechanisms impact microglia

Major PD pathomechanisms such as altered vesicular trafficking, lysosomal degradation pathways and mitochondrial function are very well characterized to affect neuronal health and survival (Vázquez-Vélez and Zoghbi, 2021). However, environmental or genetic factors which perturb these processes in neurons are likely to do the same in microglia, where each process also plays key roles. Proper sorting and trafficking of cargo through the endosomal, trans-Golgi and lysosomal pathways are critical for phagocytically active cells such as microglia (Solé-Domènech et al., 2016). Likewise, efficient lysosomal function in microglia is required to break down and recycle internalized substrates including cellular debris and aggregated proteins such as α-Synuclein (Kam et al., 2020).

Mitochondrial quality control can impact cellular energy production as well as leading to excessive ROS production from dysfunctional mitochondria (Marchi et al., 2023). As noted, the pesticide rotenone is an inhibitor of electron transfer by complex I of the mitochondrial electron transport chain, also impairing mitochondrial function and generating excess ROS (Przedborski and Ischiropoulos, 2005). Treatment of mouse primary microglia or immortalized microglia with rotenone or other transport chain inhibitors potentiates inflammatory responses, an effect mediated through intracellular ROS (Sarkar et al., 2017; Gao et al., 2013; Ye et al., 2016). Genetic inhibition of microglial complex I activity in mice has been shown to induce an inflammatory gene expression signature and impaired microglial phagocytosis, though another study highlighted a requirement for complex I activity in order to sustain inflammatory responses under certain conditions (Peruzzotti-Jametti et al., 2024; Mora-Romero et al., 2024). Beyond cellular energy production and excessive ROS, mitochondrial dysfunction can also be accompanied by the release of mtDNA, mtRNA and other mitochondrial components into the cytoplasm which act on innate immune sensors to directly activate inflammatory pathways in microglia (Marchi et al., 2023).

α-Synuclein, primarily produced by neurons, can also act on microglia. As noted, monomeric and various aggregated forms of α-Synuclein are highly pro-inflammatory to microglia, notably through activation of the NLRP3 (NLR family PYD-containing 3) inflammasome (Gao et al., 2023). Importantly, α-Synuclein also impairs microglial phagocytosis and autophagy/lysosomal degradation pathways, thus initiating a feedback loop that can promote the further accumulation of pathologic α-Synuclein species (Tu et al., 2021; Lv et al., 2023).

## Parkinson’s disease genes regulate microglia functions

While the large majority of studies examining the cellular effects of PD-associated mutations have focused on neuronal processes, a growing body of evidence indicates these mutations affect other brain cell types as well (Kam et al., 2020). Indeed, mutations in each of the 7 high penetrance PD genes have been shown to affect microglial functions in addition to their effects on neurons. A number of PD risk genes have also been found to impact microglia, however, further defining the cell types and cellular functions impacted by these risk factors remains a major area of interest that is largely unaddressed.

It is worth noting that studies using human induced pluripotent stem cell (iPSC) models have also been used to examine microglial alterations relevant to PD. iPSC-based studies are of critical importance due to the cross-species differences that exist in cell and protein function, and the limited translatability that has been found from studies using rodents and other model organisms (Granzotto et al., 2024; Penney et al., 2020). Such considerations are particularly important in the study of microglia, as they are the most divergent brain cell type between humans and rodent models in terms of gene expression and protein conservation (Pembroke et al., 2021).

### SNCA

α-Synuclein aggregation into Lewy bodies and neurites is a pathological hallmark that characterizes most familial and idiopathic PD cases alike (Calabresi et al., 2023). PD-causing SNCA mutations result either in overexpression of or increased aggregation of α-Synuclein (Przedborski, 2017). α-Synuclein protein can interact with vesicular membranes throughout the cell, and its best described endogenous functions involve the regulation of neurotransmitter vesicle trafficking and release (Calabresi et al., 2023).

While exogenous α-Synuclein (including neuron-derived) can impact microglia in multiple ways, SNCA mutations and altered expression also cell-intrinsically alter microglial properties. Primary microglia from SNCA null mice exhibited an inflammatory phenotype and impaired phagocytosis (Austin et al., 2006). Similar effects were observed in immortalized mouse microglia overexpressing the A53T mutant form of α-Synuclein (Rojanathammanee et al., 2011). Importantly, microglia-specific overexpression of wild-type α-Synuclein in mice induced a reactive and inflammatory microglial state that led to the degeneration of DA neurons (Bido et al., 2021). A recent study also used gene editing and iPSC-derived microglia to examine the effects of the A53T α-Synuclein mutation on human microglia. The authors found that the A53T mutation induced a pro-inflammatory cell state, both in monoculture and following transplantation into mouse brains, as well as evidence of oxidative damage in transplanted microglia (Krzisch et al., 2024).

### PRKN and PINK1

Parkin is an E3 ubiquitin ligase, and PINK1 a protein kinase. Together they coordinate as part of the mitochondrial quality control system to detect and tag damaged mitochondria for degradation (Vázquez-Vélez and Zoghbi, 2021). Loss of function mutations affecting either gene cause early onset forms of PD. With PINK1 kinase activity directing Parkin ubiquitin ligase activity towards mitochondria, the lack of either protein allows dysfunctional ROS producing mitochondria to persist within cells, though each protein also has additional cellular substrates (Day and Mullin, 2021). Intriguingly, one of those substrates is NLRP3. Microglia from PRKN and PINK1 knockout (KO) mice show exaggerated NLRP3 inflammasome activation in response to LPS challenge (Mouton-Liger et al., 2018; Yan et al., 2023). Indeed, PRKN heterozygous or homozygous KO mice were more sensitive to treatment with LPS, exhibiting DA neuron loss and excessive NLRP3 inflammasome activation compared to wild-type mice, and suggesting that mutations affecting the PRKN/PINK1 pathway can also modulate neurodegeneration though microglial mechanisms (Wang A. et al., 2024; Yan et al., 2023). Furthermore, neuron–microglia co-cultures made from PRKN null iPSCs exhibited a pro-inflammatory response to treatment with LPS and mtDNA (Wasner et al., 2022).

### PARK7/DJ-1

DJ-1 is a multifunctional protein with deglycase, peptidase and chaperone activity that plays numerous roles in cellular protection against oxidative stress (Lind-Holm Mogensen et al., 2023). Loss of function DJ-1 mutations cause early onset familial PD and can also alter microglia function (Lind-Holm Mogensen et al., 2023). Multiple studies of mouse microglia *in vivo* and *in vitro* have reported overactive inflammatory responses to a range of stimuli by microglia lacking functional DJ-1 (Hyeon et al., 2013; Trudler et al., 2014; Chien et al., 2016; Lin et al., 2021). Increased DA neuron death was also observed in DJ-1 KO mouse brains and neuron–microglia co-cultures (Chien et al., 2016; Lin et al., 2021). A recent study, however, using both DJ-1 KO mice and iPSC-microglia, found that microglia lacking DJ-1 exhibited abnormal responses to LPS that were less classically inflammatory than those of wild-type microglia (Lind-Holm Mogensen et al., 2024).

### GBA1

GBA1 encodes the lysosomal hydrolase glucosylceramidase, required for the breakdown of the membrane sphingolipid glucosylceramide (GC). GBA1 mutations impairing enzymatic function cause the lysosomal storage disorder Gaucher disease (GD), with patients frequently developing Parkinsonian symptoms (Huh et al., 2023). GBA1 mutations, most often heterozygous, are also a highly prevalent cause of PD (Huh et al., 2023). Impairment of GBA1 function results in the buildup of GC in lysosomes and the impairment of normal lysosomal function (Huh et al., 2023). In iPSC-derived macrophages from GD patients, GBA1 loss of function caused elevated levels of multiple pro-inflammatory cytokines under baseline conditions as well as following stimulation (Panicker et al., 2014; Serfecz et al., 2021). Furthermore, in mice lacking brain GBA1, selective microglial rescue improved survival, while microglia-specific deletion caused neurodegeneration, however, another study reported no effect after microglia-specific cKO (Boddupalli et al., 2022; Duffy et al., 2024). Importantly, GC itself has also been shown to activate microglial inflammatory responses and induce phagocytosis of neurons in mouse models (Shimizu et al., 2023).

### LRRK2

LRRK2 is a multifunctional protein kinase with a wide range of described cellular functions (Bailey and Cookson, 2024). LRRK2 protein coding mutations such as the G2019S substitution cause familial PD by either directly or indirectly increasing kinase activity and have well described effects in multiple different cell types (Bailey and Cookson, 2024). SNPs that alter LRRK2 expression are also risk factors for PD, with this risk having been attributed specifically to increased LRRK2 expression in microglia (Langston et al., 2022). In the mouse brain and primary microglia, the LRRK2 G2019S mutation impaired cellular motility, altered lysosomal trafficking and increased inflammatory responses to LPS and α-Synuclein (Choi et al., 2015; Ho et al., 2017; Russo et al., 2018; Mamais et al., 2021). Human iPSC-derived microglia carrying the G2019S mutation had altered cytokine release and lysosomal trafficking and produced neurotoxic factors that reduced neurite length in DA neurons (Mamais et al., 2021; Panagiotakopoulou et al., 2020). Moreover, the function of TNTs that form between neurons and microglia was impaired by LRRK2 mutation. LRRK2 G2019S reduced the transfer of mitochondria to neurons and of α-Synuclein to microglia in mouse models, with the latter also being impaired in iPSC-derived neuron–microglia cultures (Scheiblich et al., 2024).

### VPS35

VPS35 is a core component of the retromer complex, critical for sorting and transport of specific cargo proteins within the endo-lysosomal and trans-Golgi networks (Rowlands and Moore, 2024). The VPS35 D620N mutation causes PD, however, whether this mutation works through a gain of function, partial loss of function or dominant negative effect is not known (Rowlands and Moore, 2024). Knockdown of VPS35 in immortalized mouse microglia, however, caused an exaggerated pro-inflammatory response to LPS treatment (Yin et al., 2016). Intriguingly, microglia-specific VPS35 cKO in mice also led to increased microglial phagocytosis of synaptic proteins in the hippocampus and was associated with reduced synapse density (Appel et al., 2018). Whether DA neurons may be similarly affected by VPS35 mutations in PD has not been examined, however, multiple other mutations associated with neurodegenerative diseases, potentially including LRRK2 G2019S, appear to promote microglia-mediated synapse loss (Penney et al., 2024; Lall et al., 2021; Zhang et al., 2022; Ayala et al., 2024). Notably, a recent study also reported that the VPS35 D620N mutation increases LRRK2 kinase activity in the mouse brain and that LRRK2 kinase inhibitors could correct D620N-dependent alterations in dopamine physiology, potentially linking these two familial PD genes (Bu et al., 2023).

### Other PD genes

The S71R mutation in RAB32 was very recently identified as a cause of familial PD (Hop et al., 2024; Gustavsson et al., 2024). RAB32 is a microglia-enriched GTPase that regulates vesicle transport and plays known roles in defense against bacterial infection (Liang et al., 2012). RAB32 was also shown to physically interact with fellow PD protein LRRK2 and with the retromer complex containing VPS35, with the RAB32 S71R mutation promoting LRRK2 kinase activity (Hop et al., 2024; Gustavsson et al., 2024). RAB32 mutations have not yet been studied in microglia, or indeed in any PD context beyond the interactions with LRRK2 and VPS35, thus such studies will be of considerable interest.

A number of the nominated PD risk genes have also been shown to impact microglial functions. For instance, BAG2, CTSB, DYRK1A, FYN, GALC, GPNMB, and NOD2, among others, have been reported to regulate microglial phagocytosis, degradation pathways and/or inflammatory responses (Nalls et al., 2019; Kim et al., 2022; Ying et al., 2022; Ju et al., 2022; Panicker et al., 2019; Scott-Hewitt et al., 2018; Sun et al., 2012; Hou et al., 2024). As noted, however, the functions of most known PD risk factors have not yet been examined in microglia, nor in most other brain cell types.

## Discussion

While much is known about perturbed cellular functions and pathways in PD, the most important contributors to disease remain heavily debated (Müller-Nedebock et al., 2023). Establishing the critical cellular functions, cell types, and disease mechanisms that are impacted by the large number of relatively newly discovered PD risk genes will help to provide a fuller understanding of disease etiology. In addition, the continued refinement and wider use of human stem cell-based models is facilitating the study of human disease using human cells, bringing considerable hope for improved translatability of identified disease mechanisms and therapies.

The roles of microglia have been increasingly appreciated across the full spectrum of neurodegenerative diseases. While historically thought simply to be secondary responders to the neurodegenerative process, genetic and functional studies have demonstrated that microglia play more active roles in at least a subset of disease cases. Of particular relevance to PD, microglia can integrate pathological inputs from multiple sources: a range of environmental factors can activate microglia, hallmark protein aggregates trigger microglia inflammatory and impair homeostatic responses, and key genetic factors perturb microglial functions. Furthermore, in multiple PD-relevant experimental models, microglia-specific manipulation or microglial depletion demonstrate causal roles for these cells in driving DA neuron degeneration. Continued focus on defining the full diversity of cell types and perturbed cellular functions that contribute to neuron degeneration will lead to a more holistic understanding of disease mechanisms. We believe that such a foundation will allow for the development of more effective therapeutic approaches, potentially by targeting multiple disease processes across multiple cell types.
